# Genetic parameters of colostrum and calf serum antibodies in Swedish dairy cattle

**DOI:** 10.1186/s12711-022-00758-y

**Published:** 2022-10-22

**Authors:** Juan Cordero-Solorzano, Dirk-Jan de Koning, Madeleine Tråvén, Therese de Haan, Mathilde Jouffroy, Andrea Larsson, Aline Myrthe, Joop A. J. Arts, Henk K. Parmentier, Henk Bovenhuis, Jonas Johansson Wensman

**Affiliations:** 1 Department of Animal Breeding and Genetics, Swedish University of Agricultural Sciences, P.O. Box 7023, 750 07 Uppsala, Sweden; 2 Department of Clinical Sciences, Swedish University of Agricultural Sciences, P.O. Box 7054, 750 07 Uppsala, Sweden; 3 Animal Breeding and Genomics, Wageningen University & Research, P.O. Box 338, 6700 AH Wageningen, The Netherlands; 4 Adaptation Physiology Group, Wageningen University & Research, P.O. Box 338, 6700 AH Wageningen, The Netherlands; 5 AgroSup Dijon - National Superior Institute of Agronomic Sciences Food and the Environment, 26 Boulevard Dr Petitjean, 21079 Dijon, France; 6 Superior School of Agricultures (ESA), 55 Rue Rabelais, 49007 Angers, France; 7 Animal Health Service of Costa Rica (SENASA), P.O. Box 3-3006, 40104 Heredia, Costa Rica; 8 Department of Disease Control and Epidemiology, National Veterinary Institute, 751 89 Uppsala, Sweden

## Abstract

**Background:**

A sufficient IgG content in the colostrum is essential for the newborn calf, as it provides passive immunity which substantially affects the probability of survival during rearing. Failure of passive transfer (FPT) occurs when a calf does not absorb enough antibodies from the colostrum and is defined by an IgG concentration in calf serum lower than 10 g/L. Apart from delayed access to colostrum, FPT can be due to a low production of IgG in the mother or poor IgG absorption by the calf. The aim of this study was to estimate the genetic background of antibody levels and indicator traits for antibodies in the colostrum and calf serum, and their correlation with milk production.

**Results:**

Colostrum data were available for 1340 dairy cows with at least one calving and calf serum data were available for 886 calves from these cows. Indicator traits for antibody concentrations were estimated using refractometry (a digital Brix refractometer for colostrum and an optical refractometer for serum), and enzyme-linked immunosorbent assays (ELISA) were used to determine the levels of total IgG and natural antibodies (NAb) of various antibody isotypes in the colostrum and calf serum. Colostrum traits had heritabilities ranging from 0.16 to 0.31 with repeatabilities ranging from 0.21 to 0.55. Brix percentages had positive genetic correlations with all colostrum antibody traits including total IgG (0.68). Calf serum antibody concentrations had heritabilities ranging from 0.25 to 0.59, with a significant maternal effect accounting for 17 to 27% of the variance. When later in life calves produced their first lactation, the lactation average somatic cell score was found to be negatively correlated with NAb levels in calf serum.

**Conclusions:**

Our results suggest that antibody levels in the colostrum and calf serum can be increased by means of selection.

## Background

At birth, calves depend highly on the absorption of maternal antibodies from the colostrum to acquire passive humoral immunity and local protection of the digestive tract. If the amount of transferred antibodies is not sufficient, i.e. if 24 h after birth the levels are lower than 10 g/L for IgG or lower than 5.5 g/dL for serum total protein (STP) in the calf serum [1], failure of passive transfer (FPT) occurs. Calves with FPT have a two-fold higher risk of morbidity and death at a young age compared to calves with normal levels of serum IgG (S-IgG) [2]. Estimates of the prevalence of FPT in Swedish dairy herds range from 14% [3] to 60% [4].

Assessing colostrum quality is an important factor to prevent FPT. Radial immunodiffusion (RID) assay is the gold standard for measuring IgG directly in the colostrum or serum [5]. It requires an adequate laboratory setting, is time-consuming and relatively expensive, which make it impractical for phenotyping a large number of animals. Alternatively for a direct measurement of IgG in the colostrum or serum, a quantitative enzyme-linked immunosorbent assay (ELISA) is available, which is cheaper and marginally faster than RID but also requires an appropriate laboratory setting [6]. Colostrum quality and passive transfer can be assessed with indirect methods that measure total solid (TS) or total protein (TP), usually with a refractometer. By this technique, the refraction of light is measured in the serum or colostrum to obtain an estimation of total protein concentration. Serum total protein has been found to correlate reasonably well (r = 0.72) with the IgG level measured by RID [1]. The Brix refractometer (originally defined for sugar solutions) has also been used to evaluate colostrum quality, with correlations between Brix percentages and RID values ranging from 0.64 to 0.75 [5, 7, 8]. These studies reported cut-off points for colostrum quality at 21 to 22% that correspond to about 50 g/L of IgG, which is considered to be a sufficient concentration to transfer adequate passive immunity to the calf [5].

Colostrum quality varies largely between cows, even between animals from the same farm and breed [9]. Environmental factors explain some of this variation, but results in the literature suggest that there is an important genetic component in antibody content of the colostrum. Gilbert et al. [10] estimated a heritability of 0.4 (standard error (SE) = 0.3) for colostrum IgG. More recently, a study by Soufleri et al. [11] found a heritability of 0.27 (0.09) for Brix percentage in the colostrum of Holstein cows.

In spite of its importance for calf health, very few studies have focused on the genetics associated with the calf’s antibody uptake from the colostrum. It has been observed that even when the time of the first meal, the volume of the first meal, the colostrum antibody concentration and other variables are accounted for, a significant part of the variation in calf antibody uptake remains unexplained [12], which is likely due to genetics. Gilbert et al. [10] estimated a heritability of 0.56 (0.25) for calf serum IgG1 in 36-h-old calves using a paternal half-sib analysis accounting for the dam’s colostrum IgG1 concentration and Burton et al. [13] estimated a heritability of 0.18 (0.25) for serum IgG in 24 to 36-h-old calves using a paternal half-sib analysis. More recently, using a dataset of 366 Charolais calves, Martin et al. [14] estimated a heritability of 0.36 (0.18) for calf serum IgG at 24 to 48 h after birth.

Natural antibodies (NAb) are immunoglobulins that are produced without any antigenic stimulation by a subset of the B cells known as B-1 cells, which are part of the innate immune system [15, 16]. These antibodies likely play a role in the first defense against a variety of infectious diseases and tumors [17]. Genetic studies in dairy cattle have found associations of NAb levels with risk of mastitis [18], length of productive life [19] and lameness [20]. In poultry, serum NAb levels have been linked to survival [21] and *E. coli* resistance [22]. Antibodies are classified into three isotypes IgM, IgG, and IgA, based on their heavy chain structure. In the early stages of an infection, IgM is the first isotype produced, allowing a quick response to a variety of microbial pathogen-associated molecular patterns (PAMP) and other antigens [23]. Upon antigenic stimulation, B cells can switch isotype and differentiate into plasma cells that produce more specific IgG antibodies [24]. An important part of the humoral immune response relies on NAb, making them an interesting target for potential selection to improve disease resistance.

Understanding the genetic relationships between the antibodies in the colostrum and those in the calf serum is crucial to reduce the occurrence of FPT and improve calf health. Given the significant heritabilities reported in cattle for these traits and their association with production and health traits, they show great potential to be used in genetic evaluations. The aims of this study were to determine if there is a measurable genetic component in calf antibody uptake from the colostrum using calf serum antibody traits and STP as indicators and how these traits correlate with milk production traits later in life. Another aim was to measure the genetic components of antibody traits from the colostrum, milk and serum using Brix percentage as an indicator and how these traits correlate with milk production and calf antibody uptake.

## Methods

### Samples

The samples were collected on three experimental farms in Sweden: the Swedish Livestock Research Center Lövsta (Uppsala), Röbäcksdalen forskningsstation SITES (Umeå) and Nötcenter Viken (Falköping) from January 2015 to April 2017. An overview of the traits and number of analyzed samples per farm and breed are in Table 1. Samples arrived frozen from the farms to SLU’s Centre for Veterinary Medicine and Animal Science (VHC) in Uppsala and were kept at − 20 °C until they were analyzed, except the serum samples which were stored at − 80 °C.

**Table 1:** Overview of traits and number of analyzed samples by breed and farm

Sample type	Trait	N	Breed			Farm		
			SRB	SLB	CRB	L	R	V
Colostrum	Brix (%)	1682	870	567	245	568	225	889
	Total IgG (g/L)	686	482	204	–	504	185	–
	NAb (titer)	1519–1532	808	515	209	570	228	734
First test milk	NAb (titer)	248–272	170	102	–	272	–	–
Pre-parturition serum	NAb (titer)	321	197	124	–	321	–	–
Calf serum	STP (g/dL)	808	507	207	93	581	227	–
	S-IgG (g/L)	810	509	210	91	584	226	–
	NAb (titer)	774–827	520	211	96	594	233	–

*N* number of analyzed samples; *SRB* Swedish Red, *SLB* Swedish Holstein, *CRB* SRB/SLB crossbred; *L* Lövsta forskningscentrum, *R* Röbäcksdalen forskningsstation SITES, *V* Nötcenter Viken; *NAb* natural antibodies, *STP* serum total protein, *S-IgG* serum IgG

### Cows

In total, 1340 cows were sampled and included 682 Swedish Red (SRB), 460 Swedish Holstein (SLB) and 198 crossbreds between SLB and SRB (CRB). Parities ranged from 1 to 6 with 50% of the animals in first, 23% in second and 14% in third parity. During the sampling period, 504 cows calved twice and 29 calved three times.

Sampling of first milking colostrum was carried out by staff on the farms who recorded the time between birth and sampling for 1711 individual samples, but 29 of these were excluded due to labeling issues. In addition, pre-parturition serum and first test milk samples were obtained only from cows at the Lövsta center, among which there were 330 serum samples from January 2015 to June 2016 between days 1 and 23 before calving and 290 milk samples from February 2015 to March 2016 at 6 to 24 days in milk. All colostrum and milk samples were sent in sterile and pyrogen-free 50-mL Falcon tubes.

Information on milk production was provided by Växa Sverige for 1283 cows and comprised 305-day milk yield (kg), fat and protein percentage and lactation-average somatic cell score (LASCS) from lactations matching the corresponding colostrum sample. LASCS values were calculated from somatic cell count (SCC) measurements as described by Wijga et al. [25].

### Calves

In total, 886 calves, which had been calved by cows with a matching colostrum sample, were sampled at Lövsta and Röbäcksdalen. However, 59 calves that could not be linked to a pedigree record were excluded from the study. The remaining 827 calves included 520 SRB, 211 SLB and 96 CRB individuals. Calves were sampled between the ages of 1 and 12 days, but 93% of the samples were taken before they were 8 days old. For each calf, birth weight (measured with a scale), volume of the first meal, time of the first meal after birth, and colostrum donor of the first meal, were recorded. Neonates were separated from the dam as soon as possible after birth. At Röbäcksdalen, all calves were fed with a nipple bottle, while at Lövsta, most of them were fed with a nipple bottle except those that were too weak to suckle and thus fed by esophageal tube. All calves were given first milking colostrum as the first meal.

Sampling of blood was carried out by staff on the farms during specific days of the week due to logistics. For serum samples, blood was drawn using a vacutainer system (BD Biosciences, NJ, USA) in tubes without additives, and for DNA extraction and genotyping, anti-coagulated blood was obtained in tubes with K_3_-ethylenediaminetetraacetic acid (EDTA).

Information on first parity milk production was provided by Växa Sverige for 253 of the animals that started the study as calves and comprised 305-day cumulative milk yield (kg), fat and protein percentage and LASCS.

### Phenotypes

Analyses were made in batches at the laboratory of the Department of Clinical Sciences, SLU, except for the NAb tests, which were analyzed at the laboratory of the Adaptation Physiology Group, Wageningen University. Over the span of three years, Brix measurements on colostrum samples were performed at SLU in batches, with on average a different batch on average every six months by up to five people, including staff and students. IgG ELISA tests on calf serum and colostrum were performed by two people after completing all the sampling. Similarly, STP measurements on calf serum were performed by one person at the end of the experiment. The samples sent to the Netherlands were analyzed in two batches by two people: one in 2016 and one in 2018.

#### Brix

Colostrum samples were analyzed using a digital refractometer Atago 3810 PAL-1 (Atago, Tokyo, Japan) with automatic temperature compensation, which allows accurate measurements without recalibration at ambient temperatures between 10 and 40 °C. The refractometer was equipped with a Brix scale ranging from 0 to 53% and an accuracy of ± 0.2%. Brix percentage approximates the percentage of total solids (TS), as an estimate for IgG content. In total, 1682 individual samples were analyzed. Before use and between each sample, the digital refractometer was calibrated to 0 using demineralized water. Approximately 300 µL of each sample was measured three times to obtain an average value.

#### Serum total protein (STP)

An optical refractometer AO Veterinary Refractometer 10436 (AO Scientific Instruments, NY, USA) was used to measure TS in calf serum samples as an estimate of STP (g/dL). For the measurements, approximately 100 µL of serum were placed on the prism and the sample cover was lowered, the refractometer was then held up to a light source, and the STP value was read at the line between the light and dark areas that appeared on the scale. Each sample was measured three times to obtain an average value. In total, 808 samples were analyzed and 19 samples that presented severe hemolysis were excluded.

#### Total IgG in colostrum

Colostrum IgG level was measured only in samples with a matching calf serum sample using a commercial ELISA test (Bovine IgG ELISA Kit E-10G, Immunology Consultants Laboratory Inc, OR, USA) according to the manufacturer’s instructions. Standards and samples were run in duplicates and a four-parameter logistic regression was used to calculate the IgG concentration (g/L). Of the 827 colostrum samples with a matching calf serum sample, only 686 were analyzed due to logistical constraints. Samples were diluted at 1:400,000 but samples for which the values were high and outside the standard reference curve limits were retested at 1:800,000.

#### Total IgG in calf serum

Serum IgG concentration was determined using an ELISA kit (Bovine IgG ELISA Quantitation Set E10-118, Bethyl Laboratories Inc, TX, USA) according to the manufacturer’s instructions. Standards and samples were run in duplicates and a logit regression was used to calculate serum IgG concentration (g/L). Sera (810 samples) were diluted at 1:112,000 but samples for which the values were outside the standard reference curve limits were retested at 1:224,000 or 1:16,000 for higher or lower values, respectively. To avoid confusion with the NAb traits, these IgG measurements will be referred to as “colostrum total IgG” or simply “total IgG” for colostrum and “serum IgG” or “S-IgG” for calf serum.

#### Natural antibodies

Titers for NAb were measured in all sample types for cows (colostrum, first test milk and pre-parturition serum) and in serum for calves. Optical density (OD) of muramyl dipeptide (MDP) and keyhole limpet hemocyanin (KLH)-binding immunoglobulins of the isotypes IgM, IgA, and IgG were measured by an indirect two-step ELISA test as outlined by Ploegaert et al. [26].

Colostrum and serum samples were prediluted at 1:10 and milk samples at 1:5, with phosphate-buffered saline containing 0.05% Tween 20 (PBST pH 7.2, dilution buffer). Flat-bottomed, 96-well medium binding plates were coated with 100 μL/well of 2 μg/mL of KLH (H8283, Sigma-Aldrich), or MDP (A9519, Sigma-Aldrich), respectively, in carbonate buffer (5.3 g/L Na_2_CO_3_ and 4.2 g/L NaHCO_3_, pH 9.6). After incubation overnight at 4 °C, plates were washed with tap water containing Tween 20 and blocked with 100 μL/well of 5% normal chicken serum in PBST for one hour at room temperature.

Prediluted samples were further diluted in the antigen-coated plates with dilution buffer to 1:40, 1:160, 1:640, and 1:2560 test dilutions for colostrum and serum and 1:10, 1:20, 1:40, and 1:80 test dilutions for milk. One unrelated colostrum sample was chosen as the standard positive for all assays. Duplicates of this standard positive were stepwise 1:2 diluted with dilution buffer (8 serial dilutions from 1:20 to 1:2560) and pipetted into the antigen-coated plates. The plates were incubated for one hour and a half at room temperature.

After washing the MDP and KLH plates with PBST, they were incubated with 100 μL/well of either 1:40,000-diluted rabbit-anti-bovine IgM labelled with horseradish peroxidase (HRP) (A10-100P, Bethyl Laboratories Inc), 1:40,000-diluted sheep-anti-bovine IgG HRP (A10-118P, Bethyl Laboratories Inc) or 1:20,000-diluted rabbit-anti-bovine IgA HRP (A10-108P, Bethyl Laboratories Inc) for one hour and a half at room temperature. After washing with PBST, 100 µL substrate buffer (containing water, 10% tetramethylbenzidine (TMB) buffer [15 g/L sodium acetate and 1.43 g/L urea hydrogen peroxide; pH 5.5], and 1% tetramethylbenzidine [8 g/L TMB in DMSO]) were added and incubated for approximately 10 min at room temperature. The reaction was stopped with 50 μL of 1.25 M H_2_SO_4_. OD was measured with a Multiskan Go spectrophotometer (Thermo Scientific, MA, USA) at a wavelength of 450 nm.

Antibody titers were calculated as described by Frankena [27] and cited by de Koning et al. [28]. Optical densities of the duplicate standard positive samples were averaged for each plate. Logit values of OD per plate were calculated as follows:

$$\mathrm{logit\,OD}=\mathrm{ln}\left(\frac{\mathrm{OD}}{{\mathrm{OD}}_{\mathrm{max}}-\mathrm{OD}}\right),$$

where $\mathrm{OD}$ is the OD of a well, and ${\mathrm{OD}}_{\mathrm{max}}$ is the maximum averaged OD of the duplicate standard positive samples. The last positive well (lpw) of the averaged duplicate standard positive was set to the sixth dilution for colostrum and serum and to the seventh dilution for milk. Titers of each sample per plate were calculated as follows:

$$\mathrm{Titer}=\frac{{\mathrm{logit\,OD}}_{\mathrm{lpw}}-\left({\mathrm{logit\,OD}}_{\mathrm{sample}}-{\beta *\mathrm{log}}_{2}({\mathrm{dilution}}_{\mathrm{sample}})\right)}{\beta },$$

where ${\mathrm{logit\,OD}}_{\mathrm{lpw}}$ is the estimated logit OD at the lpw calculated with the estimated linear regression function using the log_2_-dilution value of that well, ${\mathrm{logit\,OD}}_{\mathrm{sample}}$ is the logit OD calculated of the OD closest to 50% of OD_max_ for a sample of an individual (OD_sample_), $\beta$ is a regression coefficient of the logit OD against the respective log_2_-dilution values of the averaged duplicate standard positive samples, and ${\mathrm{log}}_{2}({\mathrm{dilution}}_{\mathrm{sample}})$ is the log_2_-dilution value at which OD_sample_ occurred, as described by de Koning et al. [28].

Six traits were generated for each sample type with three isotypes measured for each of the two antigens: KLH-IgA, KLH-IgG, KLH-IgM, MDP-IgA, MDP-IgG and MDP-IgM. In total, 1510 to 1532 samples (depending on the trait) were analyzed for each colostrum trait, 248 to 272 for first test milk, 321 for pre-parturition cow serum and 774 to 827 for calf serum. A constant was added to each trait so that all the values were greater than 0.

### Statistical analysis

Variance components for the genetic effects, permanent environmental effect (colostrum traits) and maternal effects (calf serum) were estimated with an animal model using ASReml 4.1 [29]. The pedigree data used for the whole set of animals contained 29,048 records (20 generations) and was made available by Växa Sverige.

Fixed effects in Models (1) and (3) described below were selected based on their significance by an incremental Wald F statistics test including interactions. One factor that was tested but not selected was ELISA plate, which was significant by itself, but no longer significant when the storage plate effect was added. This was observed for both colostrum and calf serum traits.

For calf serum, the factors from the Wald F statistics test that were not significant include sex, ease of calving, time of first meal and whether the calf was fed with the colostrum of the mother or another cow (as a binary variable). Volume of the first meal and colostrum antibody content were combined into a single factor “absolute amount of colostral antibodies fed” with a stronger effect.

#### Models for cows

For colostrum traits, the following repeatability model was used:

1 $${y}_{ijklm}\,=\,\mu +{\beta }_{1}{C2CS}_{ijklm}+{parity}_{i}+{breed}_{j}+{HYSP}_{k}{+A}_{l}+{pe}_{m}+{e}_{ijklm},$$

where $y$ is the observation of the trait; $\mu$ is the overall mean of the trait; ${C2CS}_{ijklm}$ is a covariate describing the effect of colostrum sampling time after calving in hours; ${parity}_{i}$ represents the fixed effect of four parity classes (1, 2, 3 and 4 or more); ${breed}_{j}$ is the fixed effect of breed (SLB, SRB or CRB); ${HYSP}_{k}$ describes the fixed effect of herd-year-season of calving and sample storage plate number; ${A}_{l}$ is the random additive genetic effect assumed to be distributed as $N(\mathbf{0},\mathbf{A}{\sigma }_{a}^{2}),$ where $\mathbf{A}$ is the additive genetic relationship matrix from the pedigree and ${\sigma }_{a}^{2}$ is the additive genetic variance; ${pe}_{m}$ is the random permanent environment effect assumed to be distributed as $N(\mathbf{0},\mathbf{I}{\sigma }_{pe}^{2})$, where $\mathbf{I}$ is the identity matrix and ${\sigma }_{pe}^{2}$ is the variance of the permanent environment effect; ${e}_{ijklm}$ is the random residual effect assumed to be distributed as $N(\mathbf{0},\mathbf{I}{\sigma }_{e}^{2}),$ where $\mathbf{I}$ is the identity matrix and ${\sigma }_{e}^{2}$ is the residual variance. For colostrum IgG, a square root transformation was applied to normalize the residual distribution.

For first test milk and pre-parturition cow serum NAb traits measured on cows from the Lövsta experimental farm the following animal model was used:

2 $${y}_{ijklm}\,=\,\mu +{\beta }_{1}{C2S}_{ijklm}+{parity}_{i}+{breed}_{j}+{YS}_{k}+{Plate}_{l}{+A}_{m}+{e}_{ijklm},$$

where $y$ is the NAb titer of milk or serum; $\mu$ is the overall mean of the trait; ${C2S}_{ijklm}$ is a covariate describing the effect of sampling time in days before (serum) or after (milk) calving; ${parity}_{i}$ represents the fixed effect of four parity classes (1, 2, 3 and 4 or more); ${breed}_{j}$ is the fixed effect of breed (SLB, SRB or CRB); ${YS}_{k}$ describes the fixed effect of year-season of calving; ${Plate}_{l}$ is the fixed effect of sample storage plate number; ${A}_{m}$ the random additive genetic effect assumed to be distributed as $N(\mathbf{0},\mathbf{A}{\sigma }_{a}^{2})$, where $\mathbf{A}$ is the additive genetic relationships matrix from the pedigree and ${\sigma }_{a}^{2}$ is the additive genetic variance; ${e}_{ijklm}$, is the random residual effect assumed to be distributed as $N(\mathbf{0},\mathbf{I}{\sigma }_{e}^{2}),$ where $\mathbf{I}$ is the identity matrix and ${\sigma }_{e}^{2}$ is the residual variance. Since repeated measurements from the same animal were not available for these two traits, the permanent environment effect could not be estimated.

#### Models for calves

For calf serum traits, the following animal model was used:

3 $${y}_{ijkl}\,=\,\mu +{\beta }_{1}{COL}_{ijkl}+{\beta }_{2}{BLD}_{ijkl}+{\beta }_{3}{BWT}_{ijkl}+{breed}_{i}+{HYSP}_{j}{+A}_{k}+{m}_{l}+{e}_{ijkl},$$

where $y$ is the observation of the trait; $\mu$ is the overall mean; ${COL}_{ijkl}$ is a covariate describing the absolute amount of antibodies received from the matching colostrum trait (Brix, colostrum IgG or NAb * volume of the first meal); ${BLD}_{ijkl}$ is a covariate describing the time from calving to blood sampling in days; ${BWT}_{ijkl}$ is the covariate of calf birth weight in kg; ${breed}_{i}$ is the fixed effect of breed (SLB, SRB or CRB); ${HYSP}_{j}$ describes the fixed effect of herd-year-season of calving and sample storage plate number; ${A}_{k}$ is the random additive genetic effect assumed to be distributed as $N(\mathbf{0},\mathbf{A}{\sigma }_{a}^{2})$, where $\mathbf{A}$ is the additive genetic relationships matrix from the pedigree and ${\sigma }_{a}^{2}$ is the additive genetic variance; ${m}_{l}$ is the random maternal effect assumed to be distributed as $N(\mathbf{0},{\varvec{I}}{\sigma }_{m}^{2})$, where $\mathbf{I}$ is the identity matrix and ${\sigma }_{m}^{2}$ is the maternal variance; ${e}_{ijkl}$ is the random residual effect assumed to be distributed as $N(\mathbf{0},\mathbf{I}{\sigma }_{e}^{2})$, where $\mathbf{I}$ is the identity matrix and ${\sigma }_{e}^{2}$ is the residual variance. For calf serum IgG, a square root transformation was applied to normalize the residual distribution.

#### Estimation of heritabilities and variance proportions

Heritabilities were estimated as:

4 $${h}^{2}=\frac{{\sigma }_{a}^{2}}{{\sigma }_{p}^{2}},$$

with phenotypic variance ${\sigma }_{p}^{2}={\sigma }_{a}^{2}+{\sigma }_{pe}^{2}+{\sigma }_{e}^{2}$ for Model (1), ${\sigma }_{p}^{2}={\sigma }_{a}^{2}+{\sigma }_{e}^{2}$ for Model (2) and ${\sigma }_{p}^{2}={\sigma }_{a}^{2}+{\sigma }_{m}^{2}+{\sigma }_{e}^{2}$ for Model (3), where ${\sigma }_{a}^{2}$ is the additive genetic variance, ${\sigma }_{pe}^{2}$ the permanent environment variance, ${\sigma }_{m}^{2}$ is the maternal variance and ${\sigma }_{e}^{2}$ the residual variance.

Repeatability for colostrum traits was:

5 $$r=\frac{{\sigma }_{a}^{2}+{\sigma }_{pe}^{2}}{{\sigma }_{a}^{2}+{\sigma }_{pe}^{2}+{\sigma }_{e}^{2}},$$

where ${\sigma }_{a}^{2}$ is the additive genetic variance, ${\sigma }_{pe}^{2}$ the permanent environment variance and ${\sigma }_{e}^{2}$ the residual variance.Maternal contribution or maternal variance proportion for calf traits was estimated as:

6 $${m}^{2}=\frac{{\sigma }_{m}^{2}}{{\sigma }_{a}^{2}+{\sigma }_{m}^{2}+{\sigma }_{e}^{2}}.$$

Genetic correlations were estimated using bivariate analyses between different colostrum traits (Model 1) and between different calf serum traits (Model 3) as follows:

7 $${r}_{g}=\frac{{\sigma }_{a1, a2}}{\sqrt{({\sigma }_{a1}^{2}*{\sigma }_{a2}^{2})}},$$

where ${\sigma }_{a1}^{2}$ is the additive genetic variance for trait 1, ${\sigma }_{a2}^{2}$ the additive genetic variance for trait 2, and ${\sigma }_{a1, a2}$ the additive genetic covariance between traits 1 and 2. The same formula was applied for phenotypic correlations, substituting the additive genetic variances and covariance by the phenotypic variances and covariance.

Genetic and phenotypic correlations were also estimated between colostrum (Model 1) and calf serum traits (Model 2). A similar approach was used to estimate genetic and phenotypic correlations of colostrum traits (Model 1) with first test milk (Model 2) and pre-parturition cow serum (Model 3).

The significance of heritabilities, variance proportions and correlations were checked using a likelihood-ratio test. For ${h}^{2}$, ${m}^{2}$ and $r$, each univariate model was compared to a model in which the corresponding variance component was fixed at almost 0 (0.001). Similarly, for the correlations, each bivariate model was compared to a model in which the genetic covariance was set to almost 0 (0.001). The variance component or covariance was deemed significant if p < 0.05.

### Analysis of production traits

#### Cows

Using available data on milk production for 305 days average of milk yield, fat and protein percentage and LASCS, the genetic and phenotypic correlations of these traits with the colostrum traits were estimated.

The following repeatability model was used for milk production traits:

8 $${y}_{ijklm}\,=\,\mu +{\beta }_{1}{AFC}_{ijklm}+{parity}_{i}+{breed}_{j}+{HYS}_{k}{+A}_{l}+{pe}_{m}+{e}_{ijklm},$$

where $y$ is the observation of the milk trait; $\mu$ is the overall mean; ${AFC}_{ijklm}$ is a covariate describing the effect of age at first calving in months; ${parity}_{i}$ represents the fixed effect of four parity classes (1, 2, 3 and 4 or more); ${breed}_{j}$ is the fixed effect of breed (SLB, SRB or CRB); ${HYS}_{k}$ describes the fixed effect of herd-year-season of calving; ${A}_{l}$ is the random additive genetic effect assumed to be distributed as $N(\mathbf{0},\mathbf{A}{\sigma }_{a}^{2})$, where $\mathbf{A}$ is the additive genetic relationship matrix from the pedigree and ${\sigma }_{a}^{2}$ is the additive genetic variance; ${pe}_{m}$ is the random permanent environment effect assumed to be distributed as $N(\mathbf{0},\mathbf{I}{\sigma }_{pe}^{2})$, where $\mathbf{I}$ is the identity matrix and ${\sigma }_{pe}^{2}$ is the variance of the permanent environment effect; ${e}_{ijklm}$ is the random residual effect assumed to be distributed as $N(\mathbf{0},\mathbf{I}{\sigma }_{e}^{2})$, where $\mathbf{I}$ is the identity matrix and ${\sigma }_{e}^{2}$ is the residual variance.

A bivariate analysis with Model (1) for the colostrum traits and Model (8) for the production traits was used to estimate the correlations according to Eq. (7).

#### Calves

First parity milk production data from 253 of the calves was combined with 700 records of first parity milk data from the project on calves’ dams. The genetic and phenotypic correlations of calf serum traits with milk production traits were estimated. A bivariate analysis with Model (3) for the serum traits and a modified Model (8) removing the fixed effect of parity and the random effect of permanent environment, was used for the production traits and age at first calving (AFC) as dependent variables to estimate the correlations according to Eq. (7).

## Results

An overview of the genetic parameters for calf serum traits and for cow colostrum, milk and serum traits are in Tables 2 and 3, respectively. For calf serum traits, the heritability estimates were moderate to high, ranging from 0.25 to 0.59, except for STP, for which ${h}^{2}$ was not significantly different from 0. Maternal contributions were moderate (0.17–0.27), except for KLH-IgA, for which it was not significantly different from 0. Breed effect was only significant for STP with an effect size of 0.27 for SLB compared to SRB.

**Table 2:** Descriptive statistics, heritabilities (${h}^{2}$), maternal effects (${m}^{2}$), phenotypic variances (${\sigma }_{p}^{2}$), breed effects and number of samples for calf serum traits

Trait	Mean (SD)	Range	${h}^{2}$	${m}^{2}$	${\sigma }_{p}^{2}$	Breed^a^		N
						SLB	CRB	
STP (g/dL)	6.0 (0.7)	4.9–7.2	0.07 (0.11)	**0.22 (0.08)**	0.44 (0.03)	**0.27 (0.10)**	**0.18 (0.09)**	808
S-IgG (g/L)^b^	22.8 (12.8)	5.6–48.7	**0.25 (0.13)**	**0.27 (0.08)**	1.28 (0.08)	0.04 (0.24)	0.06 (0.18)	810
KLH-IgA	4.0 (1.3)	1.8–6.2	**0.46 (0.14)**	0.03 (0.08)	0.85 (0.05)	0.06 (0.24)	− 0.01 (0.16)	827
KLH-IgG	9.3 (1.7)	6.3–11.6	**0.26 (0.14)**	**0.37 (0.08)**	1.57 (0.10)	0.19 (0.27)	− 0.21 (0.19)	827
KLH-IgM	6.1 (1.7)	3.1–8.8	**0.23 (0.13)**	**0.23 (0.08)**	2.24 (0.13)	0.04 (0.31)	− 0.04 (0.23)	827
MDP-IgA	5.8 (1.8)	2.6–8.4	**0.43 (0.16)**	**0.17 (0.08)**	1.63 (0.10)	-0.03 (0.33)	0.16 (0.22)	774
MDP-IgG	6.7 (1.4)	4.3–9.0	**0.24 (0.14)**	**0.25 (0.08)**	1.33 (0.08)	0.14 (0.25)	− 0.10 (0.18)	821
MDP-IgM	7.8 (1.8)	4.4–10.0	**0.59 (0.16)**	**0.19 (0.08)**	1.61 (0.10)	0.08 (0.35)	0.03 (0.22)	825

Numbers in parenthesis are the standard errors

Bold characters highlight significant ${h}^{2}$, ${m}^{2}$ and breed effects

Range shows the 5th quantile and 95th quantile, respectively

The natural antibodies are coded as follows: *MDP* Muramyl dipeptide, *KLH* Keyhole limpet hemocyanin, *IgA* Immunoglobulin A, *IgG* Immunoglobulin G, *IgM* Immunoglobulin M

*N* number of analyzed samples; *STP* serum total protein, *S-IgG* serum IgG

^a^Breed effects are relative to Swedish Red (SRB)

^b^Variance components and effects were estimated using a square root transformation

**Table 3:** Descriptive statistics, heritabilities (${h}^{2}$), repeatabilities ($r$), phenotypic variances (${\sigma }_{p}^{2}$), breed effects and number of samples for cow sample type and traits

Trait	Mean (SD)	Range	${h}^{2}$	$r$	${\sigma }_{p}^{2}$	Breed^a^		N
						SLB	CRB	
Colostrum								
Brix (%)	21.9 (4.2)	14.5–28.7	**0.31 (0.06)**	**0.35 (0.04)**	15.47 (0.59)	**2.02 (0.82)**	**0.86 (0.52)**	1682
Total IgG (g/L)^b^	56.8 (26.9)	19.7–106.6	**0.20 (0.09)**	**0.21 (0.08)**	2.49 (0.14)	**0.68 (0.32)**	**-**	686
KLH-IgA	5.9 (1.3)	3.8–8.0	**0.26 (0.06)**	**0.45 (0.04)**	1.37 (0.06)	**0.84 (0.23)**	**0.41 (0.16)**	1532
KLH-IgG	8.0 (1.6)	5.1–10.3	**0.16 (0.07)**	**0.45 (0.04)**	2.10 (0.08)	**0.94 (0.24)**	**0.50 (0.18)**	1519
KLH-IgM	7.0 (1.3)	5.0–8.7	**0.24 (0.07)**	**0.40 (0.05)**	1.40 (0.06)	**0.78 (0.23)**	**0.47 (0.16)**	1532
MDP-IgA	6.4 (1.4)	4.0–8.6	**0.29 (0.07)**	**0.38 (0.05)**	1.61 (0.07)	**0.71 (0.26)**	**0.43 (0.17)**	1532
MDP-IgG	8.4 (1.8)	5.5–11.3	**0.24 (0.07)**	**0.55 (0.04)**	2.84 (0.12)	**0.89 (0.33)**	**0.61 (0.22)**	1532
MDP-IgM	9.1 (1.4)	6.5–10.9	**0.22 (0.06)**	**0.37 (0.05)**	1.78 (0.07)	**0.80 (0.25)**	**0.60 (0.17)**	1532
First test milk								
KLH-IgA	2.1 (1.4)	1.2–5.2	0.03 (0.17)	–	1.53 (0.14)	**0.46 (0.18)**	–	272
KLH-IgG	3.1 (1.0)	1.6–4.9	**0.33 (0.17)**	–	0.95 (0.09)	**0.48 (0.26)**	–	272
KLH-IgM	2.4 (0.9)	1.3–3.8	0.00 (0.00)	–	0.72 (0.06)	**0.36 (0.11)**	–	272
MDP-IgA	2.9 (1.3)	1.3–5.4	0.15 (0.20)	–	1.43 (0.14)	**0.45 (0.25)**	–	248
MDP-IgG	2.9 (0.8)	1.7–4.4	0.00 (0.00)	–	0.66 (0.06)	**0.23 (0.11)**	–	262
MDP-IgM	3.4 (1.2)	1.8–5.7	0.03 (0.15)	–	0.89 (0.08)	0.18 (0.14)	–	268
Pre-parturition serum								
KLH-IgA	2.6 (0.7)	1.7–3.7	**0.64 (0.16)**	–	0.36 (0.03)	0.29 (0.20)	–	321
KLH-IgG	4.8 (1.3)	2.7–7.2	**0.52 (0.16)**	–	1.38 (0.12)	**0.83 (0.36)**	–	321
KLH-IgM	7.0 (1.1)	5.4–8.9	**0.37 (0.17)**	–	1.14 (0.10)	0.15 (0.29)	–	321
MDP-IgA	3.3 (1.0)	1.6–4.8	**0.52 (0.15)**	–	0.90 (0.08)	**0.48 (0.29)**	–	320
MDP-IgG	3.1 (1.1)	1.5–5.0	0.25 (0.14)	–	0.93 (0.08)	**0.44 (0.22)**	–	321
MDP-IgM	3.4 (0.9)	2.0–4.9	**0.40 (0.16)**	–	0.66 (0.06)	0.07 (0.22)	–	321

Numbers in parenthesis are the standard errors

Bold characters highlight significant ${h}^{2}$, $r$ and breed effects

Range shows the 5th quantile and 95th quantile, respectively

The natural antibodies are coded as follows: *MDP* Muramyl dipeptide, *KLH* Keyhole limpet hemocyanin, *IgA* Immunoglobulin A, *IgG* Immunoglobulin G, *IgM* Immunoglobulin M

*N* number of analyzed samples

^a^Breed effects are relative to Swedish Red (SRB)

^b^Variance components and effects were estimated using a square root transformation

The heritability estimates for colostrum traits were moderate, ranging from 0.16 to 0.31 and the repeatabilities were moderate to high (0.21 to 0.55). Breed effects were significant and consistently higher for SLB and the CRB crossbreds compared to SRB. The effect of breed varied among traits, but the difference between SRB and SLB was about half a standard deviation in most cases. In the case of first test milk, only KLH-IgG had a significant heritability estimate (0.33) but breed effects were significant for all but one trait, with the difference between breeds being approximately one third of a standard deviation. The heritabilities estimated for the pre-parturition serum trait were moderate to high (0.37–0.64), except for MDP-IgG, which was not significant. SLB had a significantly stronger effect on three traits: KLH-IgG, MDP-IgG and KLH-IgM.

**Fig. 1:**
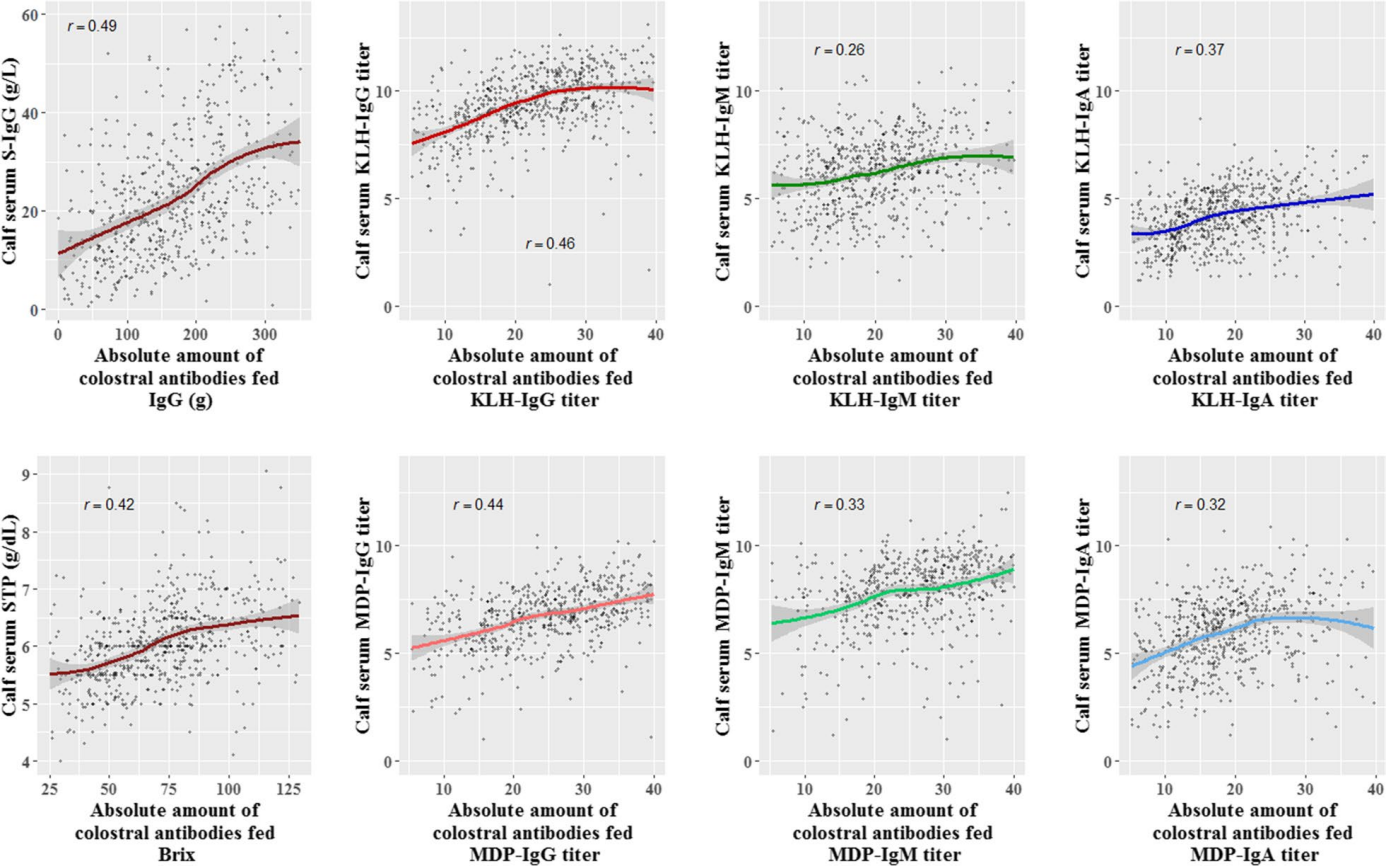
Scatterplots of each calf serum trait vs. its matching trait of absolute amount of colostral antibodies fed. For each plot, curves were constructed using a local regression function (LOESS) and Pearson correlation (r) values were estimated to visualize the relationship between the amount of antibodies received from the cows’ colostrum versus the resulting amount of antibodies in the calves’ sera (e.g. colostrum KLH-IgG vs calf serum KLH-IgG)

Figure 1 shows the plots for each calf serum trait versus its matching absolute amount of colostrum antibodies fed. Scatter plots show a fairly linear relationship between traits that seems marginally steeper for Brix percentages and IgG (0.42 to 0.49) compared to IgM and IgA (0.26–0.37).

**Fig. 2:**
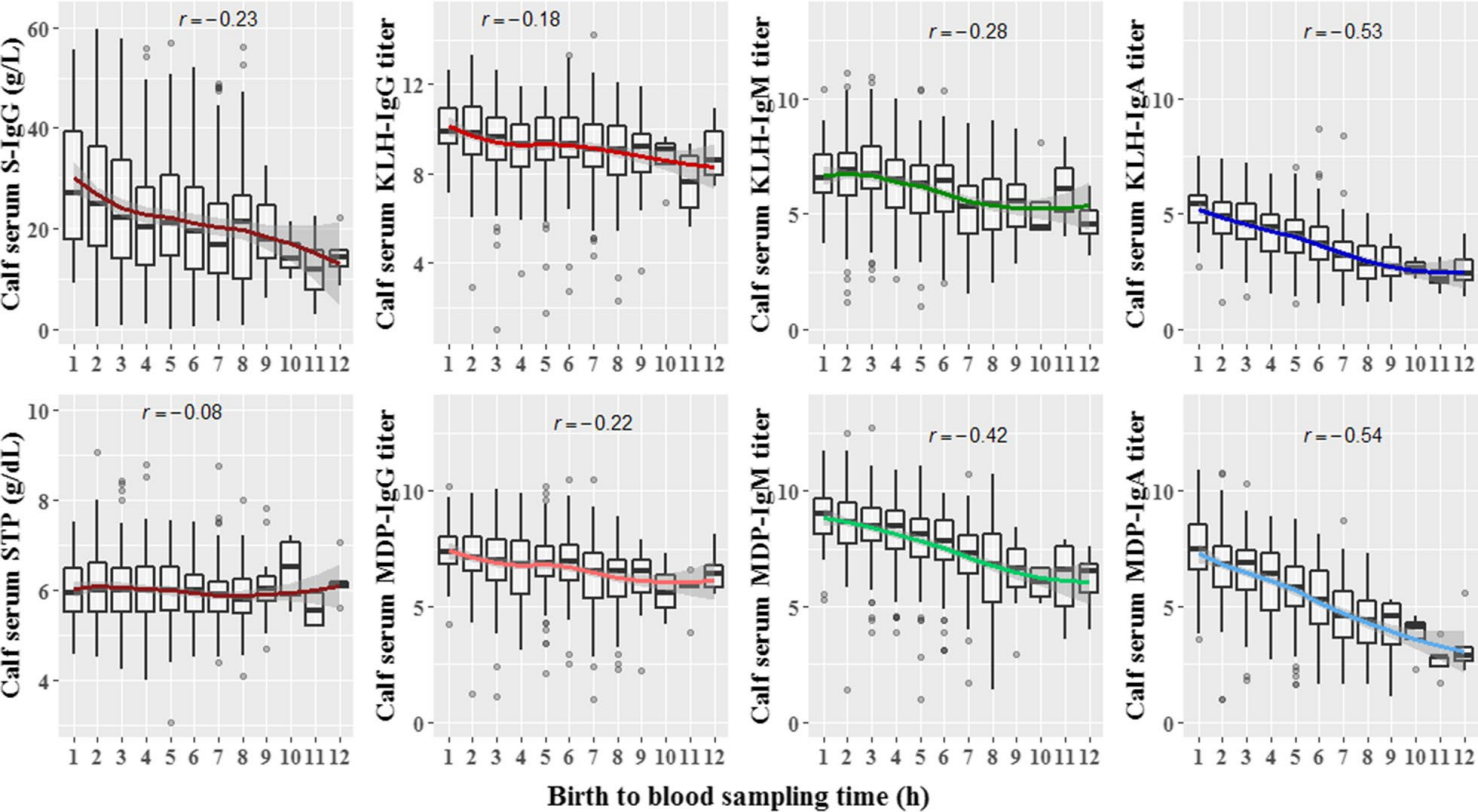
Scatterplots of each calf serum trait vs birth to blood sampling time. For each plot, curves were constructed using a local regression function (LOESS) and Pearson correlation (r) values were estimated to visualize the effect of sampling time on each calf serum trait

Time of calving to blood sampling (calves) had a linear relationship with all the calf serum traits and varying slopes depending on the trait. Figure 2 shows the plots for each trait versus the time of blood sampling in days. STP and IgG traits have a slightly lower correlation (− 0.08 to − 0.23) compared to IgM and IgA (− 0.28 to − 0.54).

For the colostrum traits, the genetic and phenotypic correlations are in Table 4. All significant genetic correlations were positive and ranged from 0.49 to 0.97, and likewise, all the phenotypic correlations were positive and ranged from 0.33 to 0.79. Remarkably, Brix percentage was positively correlated with all the traits, including total IgG.

**Table 4:** Estimated genetic correlations (below diagonal) and phenotypic correlations (above diagonal) between colostrum traits

Trait	Brix	T-IgG^a^	KLH-IgA	KLH-IgG	KLH-IgM	MDP-IgA	MDP-IgG	MDP-IgM
Brix (%)	–	0.70 (0.02)	0.52 (0.02)	0.51 (0.02)	0.60 (0.02)	0.48 (0.02)	0.45 (0.02)	0.58 (0.02)
T-IgG^2^	**0.68 (0.14)**	-	0.37 (0.04)	0.51 (0.03)	0.48 (0.03)	0.33 (0.04)	0.35 (0.04)	0.39 (0.03)
KLH-IgA	**0.71 (0.10)**	0.23 (0.26)	–	0.55 (0.02)	0.78 (0.01)	0.68 (0.01)	0.45 (0.02)	0.67 (0.02)
KLH-IgG	**0.66 (0.14)**	0.93 (0.23)^b^	0.39 (0.21)^b^	–	0.57 (0.02)	0.40 (0.02)	0.56 (0.02)	0.49 (0.02)
KLH-IgM	**0.73 (0.09)**	0.22 (0.25)	**0.85 (0.06)**	0.32 (0.23)	-–	0.67 (0.02)	0.49 (0.02)	0.79 (0.01)
MDP-IgA	**0.52 (0.13)**	0.05 (0.28)	**0.92 (0.06)**	0.13 (0.24)	**0.81 (0.09)**	–	0.41 (0.02)	0.67 (0.02)
MDP-IgG	**0.49 (0.14)**	0.33 (0.26)	**0.56 (0.15)**	**0.76 (0.15)**	0.37 (0.18)	0.44 (0.17)^b^	–	0.53 (0.02)
MDP-IgM	**0.68 (0.10)**	0.13 (0.27)	**0.82 (0.08)**	0.10 (0.29)	**0.97 (0.04)**	**0.92 (0.08)**	0.34 (0.19)	–

Numbers in parenthesis are the standard errors

Bold characters highlight significant genetic correlations

The natural antibodies are coded as follows: *MDP* Muramyl dipeptide, *KLH* Keyhole limpet hemocyanin, *IgA* Immunoglobulin A, *IgG* Immunoglobulin G, *IgM* Immunoglobulin M

^a^Variance components for correlations were estimated using a square root transformation

^b^Suggestive significance (0.05 < *p-value* < 0.10)

For the calf serum traits, the genetic and phenotypic correlations are in Table 5. Estimated phenotypic correlations were all positive and ranged from 0.37 to 0.77. Unlike the Brix percentages for the colostrum traits, here the indicator trait STP did not show significant genetic correlations with any of the other traits. Significant genetic correlations ranged from 0.62 to 0.96 and were mainly observed for MDP-IgA and MDP-IgM with the other traits, including S-IgG (0.87 and 0.81, respectively).

**Table 5:** Estimated genetic correlations (below diagonal) and phenotypic correlations (above diagonal) between calf serum traits

Trait	STP	S-IgG^a^	KLH-IgA	KLH-IgG	KLH-IgM	MDP-IgA	MDP-IgG	MDP-IgM
STP	–	0.67 (0.02)	0.49 (0.03)	0.45 (0.04)	0.37 (0.04)	0.48 (0.03)	0.43 (0.04)	0.53 (0.03)
S-IgG^a^	0.91 (0.18)	–	0.57 (0.03)	0.59 (0.03)	0.40 (0.04)	0.60 (0.03)	0.62 (0.03)	0.61 (0.03)
KLH-IgA	0.61 (0.25)	0.70 (0.16)^b^	–	0.44 (0.04)	0.53 (0.03)	0.76 (0.02)	0.49 (0.03)	0.71 (0.02)
KLH-IgG	0.71 (0.46)	0.74 (0.20)	0.23 (0.25)	–	0.40 (0.04)	0.53 (0.03)	0.66 (0.03)	0.54 (0.03)
KLH-IgM	0.47 (0.52)	0.59 (0.28)	0.80 (0.14)^b^	0.61 (0.28)	–	0.51 (0.03)	0.39 (0.04)	0.62 (0.03)
MDP-IgA	0.67 (0.31)	**0.87 (0.13)**	**0.91 (0.08)**	0.86 (0.14)^b^	0.90 (0.17)^b^	–	0.59 (0.03)	0.77 (0.02)
MDP-IgG	0.83 (0.35)	0.90 (0.15)^b^	0.80 (0.16)^b^	0.87 (0.13)^b^	0.77 (0.25)	0.99 (0.11)	–	0.49 (0.03)
MDP-IgM	0.83 (0.26)	**0.81 (0.12)**	**0.84 (0.09)**	**0.62 (0.18)**	**0.91 (0.11)**	**0.96 (0.06)**	0.77 (0.02)^b^	–

Numbers in parenthesis are the standard errors

Bold characters highlight significant genetic correlations

The natural antibodies are coded as follows: *MDP* Muramyl dipeptide, *KLH* Keyhole limpet hemocyanin, *IgA* Immunoglobulin A, *IgG* Immunoglobulin G, *IgM* Immunoglobulin M

*STP* serum total protein, *S-IgG* serum IgG

^a^Variance components for correlations were estimated using a square root transformation

^b^Suggestive significance (0.05 < *p-value* < 0.10)

Estimated correlations between the colostrum and calf serum traits are in Table 6. There were no significant genetic correlations between STP or S-IgG with Brix percentage or colostrum total IgG, but the phenotypic correlations were positive and ranged from 0.17 to 0.26. Only IgA and IgM NAb showed significant genetic correlations between each other, which ranged from 0.66 to 0.99.

**Table 6:** Genetic and phenotypic correlations between colostrum traits (vertical) and calf serum traits (horizontal)

Trait	STP	S-IgG^a^	KLH-IgA	KLH-IgG	KLH-IgM	MDP-IgA	MDP-IgG	MDP-IgM
Phenotypic correlations								
Brix (%)	0.17 (0.04)	0.20 (0.04)	0.14 (0.05)	0.14 (0.05)	0.10 (0.05)	0.14 (0.05)	0.10 (0.05)	0.21 (0.04)
T-IgG^a^	0.26 (0.04)	0.26 (0.04)	0.13 (0.04)	0.13 (0.04)	0.07 (0.04)	0.13 (0.05)	0.14 (0.04)	0.17 (0.04)
KLH-IgA	0.08 (0.04)	0.13 (0.04)	0.39 (0.04)	0.21 (0.04)	0.26 (0.04)	0.32 (0.04)	0.20 (0.04)	0.33 (0.04)
KLH-IgG	0.09 (0.05)	0.17 (0.04)	0.15 (0.04)	0.53 (0.03)	0.09 (0.05)	0.09 (0.05)	0.28 (0.04)	0.14 (0.05)
KLH-IgM	0.12 (0.04)	0.16 (0.04)	0.30 (0.04)	0.14 (0.04)	0.25 (0.04)	0.34 (0.04)	0.16 (0.04)	0.40 (0.04)
MDP-IgA	0.08 (0.05)	0.12 (0.05)	0.27 (0.04)	0.14 (0.04)	0.19 (0.04)	0.35 (0.04)	0.19 (0.04)	0.31 (0.04)
MDP-IgG	0.04 (0.04)	0.07 (0.04)	0.13 (0.04)	0.13 (0.04)	0.04 (0.04)	0.06 (0.05)	0.43 (0.04)	0.11 (0.04)
MDP-IgM	0.13 (0.04)	0.13 (0.04)	0.26 (0.04)	0.14 (0.04)	0.25 (0.04)	0.29 (0.04)	0.12 (0.04)	0.37 (0.04)
Genetic correlations								
Brix	0.54 (0.38)	0.61 (0.54)	0.39 (0.22)	0.70 (0.61)	− 0.02 (0.32)	0.31 (0.22)	NE	0.40 (0.19)
T-IgG^a^	0.95 (0.32)	0.82 (0.52)	0.43 (0.28)	0.86 (0.63)	− 0.08 (0.40)	0.18 (0.30)	0.88 (0.66)	0.48 (0.23)
KLH-IgA	0.58 (0.44)	0.51 (0.69)	**0.94 (0.18)**	0.23 (0.84)	0.70 (0.27)^b^	**0.71 (0.19)**	0.85 (0.50)	**0.78 (0.15)**
KLH-IgG	0.60 (0.42)	0.77 (0.59)	0.11 (0.40)	0.99 (0.42)	− 0.51 (0.54)	-0.31 (1.61)	0.93 (0.38)	− 0.02 (0.32)
KLH-IgM	0.75 (0.52)	0.95 (0.89)	**0.98 (0.18)**	0.34 (0.74)	0.67 (0.28)^b^	**0.67 (0.18)**	0.84 (0.59)	**0.91 (0.13)**
MDP-IgA	0.52 (0.52)	0.68 (0.96)	**0.99 (0.22)**	0.36 (0.61)	0.48 (0.30)	**0.65 (0.17)**	0.90 (0.65)	**0.77 (0.17)**
MDP-IgG	0.30 (0.44)	0.18 (0.65)	0.51 (0.31)	-0.16 (0.76)	0.03 (0.34)	0.29 (0.34)	0.91 (0.45)	0.13 (0.26)
MDP-IgM	0.99 (0.72)	0.92 (1.06)	**0.98 (0.18)**	0.43 (0.64)	**0.89 (0.27)**	**0.96 (0.29)**	0.60 (0.62)	**0.99 (0.23)**

Numbers in parenthesis are the standard errors

Bold characters highlight significant genetic correlations

The natural antibodies are coded as follows: *MDP* Muramyl dipeptide, *KLH* Keyhole limpet hemocyanin, *IgA* Immunoglobulin A, *IgG* Immunoglobulin G, *IgM* Immunoglobulin M

*T-IgG* colostrum IgG; *NE* not estimable

^a^Variance components for correlations were estimated using a square root transformation

^b^Suggestive significance (0.05 < *p-value* < 0.10)

Correlations of colostrum traits with NAb of first test milk are in Table 7. Brix percentage and colostrum IgG did not have significant genetic correlations with first test milk NAb, pre-parturition NAb, and milk production traits and the phenotypic correlations ranged from − 0.01–0.07 (data not shown). Only colostrum NAb had significant correlations with first test milk and pre-parturition NAb that ranged from 0.63 to 0.98. Milk production traits for 305d lactation including milk yield (kg), LASCS, fat percentage and protein percentage showed a positive trend with colostrum traits, however, none of these traits were significantly correlated (Table 8).

**Table 7:** Genetic correlations of colostrum traits (horizontal) with first test milk, pre-parturition serum traits and milk production traits

Trait	Brix	T-IgG^a^	KLH-IgA	KLH-IgG	KLH-IgM	MDP-IgA	MDP-IgG	MDP-IgM
First test milk								
KLH-IgA	0.14 (0.39)	0.43 (0.55)	− 0.54 (0.51)	0.93 (0.51)	− 0.45 (0.48)	− 0.21 (0.41)	**0.86 (0.46)**	− 0.07 (0.44)
KLH-IgG	0.66 (0.37)	0.26 (0.43)	0.47 (0.36)	0.79 (0.36)	0.58 (0.33)	0.41 (0.35)	**0.98 (0.37)**	0.43 (0.34)
KLH-IgM	0.83 (0.84)	0.68 (0.89)	0.99 (0.75)	0.78 (0.40)	**0.87 (0.20)**	**0.84 (0.24)**	**0.80 (0.30)**	**0.97 (0.21)**
MDP-IgA	0.02 (0.37)	− 0.22 (0.53)	0.59 (0.35)	0.82 (0.71)	0.50 (0.43)	0.59 (0.56)	1.00 (0.70)	0.47 (0.45)
MDP-IgG	0.86 (0.64)	0.61 (1.25)	0.83 (0.49)	0.83 (0.64)	0.75 (0.28)^b^	0.59 (0.33)	**0.97 (0.30)**	0.59 (0.67)
MDP-IgM	− 0.25 (4.68)	− 0.24 (4.79)	1.00 (0.99)	NE	**0.98 (0.29)**	0.72 (0.44)	NE	0.92 (1.40)
Pre-parturition serum								
KLH-IgA	− 0.21 (0.23)	− 0.13 (0.30)	0.40 (0.23)	0.20 (0.32)	**0.63 (0.23)**	**0.92 (0.17)**	0.55 (0.26)^b^	**0.80 (0.19)**
KLH-IgG	− 0.12 (0.23)	0.30 (0.31)	− 0.02 (0.25)	0.60 (0.36)	− 0.40 (0.29)	− 0.22 (0.26)	0.43 (0.26)	− 0.44 (0.26)
KLH-IgM	− 0.19 (0.32)	− 0.59 (0.42)	0.48 (0.36)	0.45 (0.49)	0.58 (0.38)	0.94 (0.52)^b^	0.45 (0.39)	**0.85 (0.33)**
MDP-IgA	− 0.28 (0.23)	0.03 (0.31)	0.45 (0.24)	0.23 (0.33)	0.39 (0.25)	**0.79 (0.21)**	**0.98 (0.24)**	**0.68 (0.24)**
MDP-IgG	0.04 (0.33)	0.35 (0.43)	**0.70 (0.32)**	**0.99 (0.35)**	− 0.03 (0.35)	0.41 (0.31)	**0.82 (0.35)**	− 0.16 (0.37)
MDP-IgM	− 0.43 (0.29)	− 0.30 (0.35)	0.43 (0.30)	− 0.04 (0.44)	0.36 (0.31)	**0.89 (0.30)**	0.23 (0.33)	**0.79 (0.31)**
Milk production								
Milk yield	0.25 (0.17)	− 0.02 (0.28)	0.04 (0.18)	0.36 (0.26)	− 0.07 (0.19)	0.12 (0.18)	0.15 (0.20)	− 0.10 (0.20)
LASCS	NE	0.03 (0.29)	NE	NE	NE	− 0.42 (0.87)	0.21 (0.27)	− 0.15 (0.24)
Fat %	− 0.15 (0.13)	0.15 (0.21)	NE	NE	NE	0.00 (0.14)	0.09 (0.15)	0.08 (0.15)
Protein %	0.00 (0.12)	0.23 (0.20)	NE	NE	NE	0.08 (0.13)	0.14 (0.14)	0.13 (0.14)

Numbers in parenthesis are the standard errors

Bold characters highlight significant genetic correlations

The natural antibodies are coded as follows: *MDP* Muramyl dipeptide, *KLH* Keyhole limpet hemocyanin, *IgA* Immunoglobulin A, *IgG* Immunoglobulin G, *IgM* Immunoglobulin M

*LASCS* lactation average somatic cell score; *NE* not estimable

^a^Variance components for correlations were estimated using a square root transformation

^b^Suggestive significance (0.05 < *p-value* < 0.10)

**Table 8:** Genetic and phenotypic correlations of calf serum traits with milk production traits

Trait	STP	S-IgG^a^	KLH-IgA	KLH-IgG	KLH-IgM	MDP-IgA	MDP-IgG	MDP-IgM
Phenotypic correlations								
AFC	− 0.04 (0.08)	0.03 (0.08)	− 0.13 (0.08)	− 0.16 (0.08)	0.03 (0.08)	0.05 (0.08)	− 0.01 (0.08)	− 0.11 (0.08)
Fat %	− 0.12 (0.07)	− 0.02 (0.07)	− 0.02 (0.07)	− 0.09 (0.07)	− 0.03 (0.07)	− 0.08 (0.07)	− 0.04 (0.07)	− 0.05 (0.07)
Milk yield	− 0.03 (0.07)	− 0.06 (0.08)	− 0.04 (0.07)	− 0.07 (0.07)	− 0.09 (0.08)	− 0.11 (0.07)	− 0.05 (0.08)	− 0.11 (0.08)
Protein %	0.02 (0.07)	0.06 (0.07)	0.02 (0.07)	− 0.01 (0.07)	0.06 (0.07)	0.05 (0.07)	0.05 (0.07)	0.02 (0.07)
LASCS	− 0.09 (0.07)	− 0.12 (0.07)	0.01 (0.07)	0.05 (0.07)	− 0.12 (0.07)	− 0.19 (0.07)	− 0.10 (0.07)	0.07 (0.07)
Genetic correlations								
AFC	− 0.18 (0.25)	0.09 (0.18)	− 0.27 (0.16)	− 0.44 (0.18)^b^	− 0.06 (0.19)	− 0.02 (0.14)	− 0.09 (0.16)	− 0.23 (0.16)
Fat %	− 0.18 (0.22)	0.00 (0.18)	− 0.14 (0.16)	− 0.08 (0.17)	0.06 (0.19)	− 0.13 (0.14)	− 0.16 (0.17)	− 0.11 (0.15)
Milk yield	− 0.38 (0.27)	− 0.20 (0.20)	0.00 (0.18)	0.06 (0.20)	− 0.20 (0.21)	− 0.32 (0.16)	− 0.16 (0.19)	− 0.28 (0.17)
Protein %	0.21 (0.21)	0.21 (0.17)	− 0.06 (0.16)	− 0.16 (0.18)	0.06 (0.18)	0.10 (0.14)	0.18 (0.16)	0.12 (0.15)
LASCS	− 0.52 (0.33)	− 0.62 (0.26)^b^	− 0.38 (0.25)	− 0.63 (0.26)^b^	**− 0.98 (0.26)**	**− 0.66 (0.22)**	− 0.74 (0.30)^b^	**− 0.86 (0.23)**

Numbers in parenthesis are the standard errors

Bold characters highlight significant genetic correlations

*AFC* age of first calving, *LASCS* lactation average somatic cell score, *STP* serum total protein, *S-IgG* serum IgG

The natural antibodies are coded as follows: *MDP* Muramyl dipeptide, *KLH* Keyhole limpet hemocyanin, *IgA* Immunoglobulin A, *IgG* Immunoglobulin G, *IgM* Immunoglobulin M

^a^Variance components for correlations were estimated using a square root transformation

^b^Suggestive significance (0.05 < *p-value* < 0.10)

## Discussion

In this study, we found a significant genetic contribution to the variation of most of the traits for both the colostrum and calf serum and significant genetic correlations between these traits. In addition, variation of most of the calf serum traits was substantially affected by environmental differences between mothers.

### Heritabilities

Two indicator traits were analyzed: Brix percentage for colostrum and STP for calf serum. They both approximate antibody content by quantifying total solids (TS) and provide methodologically simpler but less accurate measurements compared to ELISA or radial immunodiffusion (RID). Brix percentage had a moderate heritability (0.31), which was similar to the value reported (0.27) in a previous study on colostrum [11]. In contrast, the estimated heritability for STP was not significant, which could be due to the variation of the other molecules that are co-measured, thus masking the genetic variance of the antibody level.

Heritability estimates for IgG concentration (g/L) in the colostrum and calf serum were 0.20 and 0.25, respectively. In both cases, a square root transformation was applied to normalize the residual distribution. A previous study reported a heritability of 0.10 for colostrum IgG in Irish Holstein and crossbreds [30]. Although this difference between the two studies can be explained by the different populations analyzed and by other factors such as the phenotyping procedure or model used, both studies suggest that breeding for higher colostrum IgG levels is indeed possible.

In the case of calf serum, our results concur with the findings of Gilbert et al. [10] and Martin et al. [14] who estimated significant heritabilities for S-IgG (0.56 and 0.36, respectively), confirming that it is possible to also breed for increased antibody uptake from colostrum by calves. It is worth noting that these two studies found large standard errors for the heritabilities (0.25 and 0.18, respectively), as observed in other similar studies, which may be due to the use of a small number of animals (< 400). Performing ELISA or RID on a large number of samples is labor-intensive and requires specialized equipment. These values cannot be measured directly on the farm, so the samples need to be preserved and taken to a laboratory. Thus, most of the studies include only a small number of samples, and to the best of our knowledge, our study is the first one to analyze antibodies in the colostrum and calf serum on such a large number of samples. This is also why Brix percentage is important as a proxy for these traits; indeed, as it is easily measured compared to performing ELISA tests, it allows for mass phenotyping and could be implemented in selection schemes.

Two antigens were used to measure NAb: KLH and MDP. The latter is a microbial pathogen-associated molecular pattern (PAMP) that comprises the minimal peptidoglycan (PGN) motif common to both Gram-positive and Gram-negative bacteria [31] such as *Escherichia coli* and *Staphylococcus aureus*, which are ubiquitous in most environments. On one hand, in previous studies, antibodies to PGN have been regarded as NAb that reflect an animal’s innate humoral response [32, 33]. However, given the universal presence of MDP (PGN), it is reasonable to assume that the measurements for this antigen could partly represent antibodies produced by the cows as part of the adaptive immune response to bacteria in the environment. On the other hand, KLH is considered as a true measure of NAb, since cows are not normally exposed to this antigen [34]. For colostrum, all heritability estimates for NAb traits were significant and for five of the six traits, they ranged from 0.22 to 0.29, except for KLH-IgG (0.16). This range of values is within that previously reported for cow serum and milk [32, 35]. There were no major differences between heritability estimates for isotypes except for KLH-IgG. In the case of calf serum NAb, estimates of heritability ranged from 0.23 to 0.59. NAb of the IgA isotype had almost identical heritabilities (0.43 and 0.46) which was also observed for IgG (0.24 and 0.26). However, there was a large difference in heritability between KLH-IgM (0.23) and MDP-IgM (0.59). Given their size, IgA and IgG may be absorbed in a passive way regardless of the antigens to which they bind (or not), but since the IgM molecules are much larger than IgA and IgG, different transportation mechanisms may be involved that are influenced by the type of antigen that the antibodies bind to.

In the case of pre-parturition serum, in spite of the large standard errors, heritability estimates were significant except for MDP-IgG. To our knowledge, heritability estimates have not been reported for MDP-IgG in bovine serum, but a previous study in milk [32] estimated a value of 0.15 for PGN-IgG1. For first test milk, NAb heritability estimates were not significant for most traits (standard errors ranged from 0.15 to 0.20), most likely due to the mechanisms involved in the transportation of NAb from the bloodstream into the mammary gland and to the limited sample size. Other studies have estimated heritabilities for KLH NAb of different isotypes in milk [32, 35] with values ranging from 0.08 to 0.48.

Our results show moderate heritabilities for colostrum traits, including Brix percentage (0.31) as an indicator. Brix refractometry has the advantage of being easier to perform than an ELISA or RID and can potentially be implemented on farms for the routine assessment of colostrum quality. Colostrum is milked manually, without milking robots, so even if the Brix refractometer could be integrated in the routine milking systems, it would not work for colostrum. However, collecting colostrum samples for Brix analysis requires limited additional labor at the farm level. Such samples need to be collected only once per lactation for each cow and can be stored in a − 20 °C freezer until the analyses are performed. Ideally, farms can purchase or be provided with low-cost Brix refractometers, so they can perform the measurements themselves without sending them to another farm (which can be a biosafety risk) or to a laboratory with specialized equipment. Brix percentages would then be recorded and evaluated for both feeding and breeding purposes.

For calf traits, heritability estimates were moderate to high, which indicate that there is an important genetic component for these traits, and that the occurrence of FPT can potentially be reduced through genetic selection. Unfortunately, the heritability for STP, the indicator trait for calf serum, was not significant.

Providing a practical way to measure absorption of colostrum antibodies by the calf is critical to effectively implement this trait in a breeding program. For future studies, it would be interesting to analyze calf serum using a Brix refractometer since some studies show a high correlation with serum IgG [36].

### Repeatabilities (colostrum)

NAb in the colostrum had notably higher permanent environment effects (0.10–0.31) than Brix percentage (0.04) and total IgG (0.01) which made their repeatabilities proportionally higher. In this case, the permanent environment effect was the effect of repeated measures from the same cow in different parities: the more the measurements correlate across parities, the stronger the effect. The observed differences are probably due to an accumulated exposure to antigens in older cows, which generates more specific antibodies that get transferred from the serum to colostrum [30], resulting in reduced repeatabilities for Brix percentage and total IgG. However, the amount of NAb remains mainly constant throughout life [37] so repeatabilities are higher.

### Maternal contribution (calf serum)

The maternal contribution to variation in calf serum traits ranged from 17 to 37% of the variance, except for KLH-IgA, which showed no significant maternal effect. In this case, the maternal effect was the non-genetic contribution of the dam across different calvings on calf serum traits, thus the more similar the measurements between maternal siblings, the stronger the effect. For calf serum antibodies, we believe that the greatest (non-genetic) maternal contribution may come from the colostrum, and since we accounted for colostrum antibodies in the model, other colostrum components could be influencing how well the calf absorbs antibodies. Among the components of the colostrum are fat, proteins, peptides, non-protein nitrogen, vitamins and minerals, hormones, growth factors, cytokines and nucleotides [38]. It seems plausible that some of these components differ among colostrum samples from different cows and that they directly or indirectly affect antibody uptake. One example is lactoferrin, an iron-sequestering glycoprotein with antimicrobial, anti-inflammatory, and anti-oxidative properties [39]. By inhibiting bacterial growth, it might indirectly influence IgG uptake, since antibodies such as NAb will not bind these bacteria and will be available for uptake.

Apart from the colostrum, there is increasing evidence that the calf intestine is not sterile until birth. A study by Alipour et al. [40] found a low-abundant microbiota in rectal meconium and mucosa of calves sampled at birth that resembled the composition of dam oral microbiota, but included typical intestinal taxa. The exact mechanism of how these bacteria colonize in utero is not clear, but these results suggest another source of maternal effects that could impact antibody uptake in the calf.

### Other effects

Our results show that the effect of breed is slightly larger for SLB than for SRB or CRB in the colostrum, first test milk and pre-parturition serum traits. Different sample types were analyzed by different techniques, but in all cases SLB showed higher values. However, even for statistically significant effects, most *p-values* were barely lower than 0.05. Holsteins are generally assumed to have a colostrum of poorer quality, but several studies have found non-significant differences between Holstein and other breeds concerning colostrum IgG [41–43]. Breed effects were not significant for the calf serum traits except for STP, which showed the same pattern as in the colostrum.

Time of the first meal is a critical factor to avoid FPT [44], but in our case it was not significant. This is probably due to the fact that feedings were done within the appropriate window of time, since 95% of the first meals were given less than six hours after birth (not shown), which is the cut-off point for optimal feeding time [45] and well below the 24 h cut-off.

Sampling time after birth showed a stronger negative correlation with the IgM and IgA traits than with the IgG traits and STP. This is most likely because IgG has a half-life of 28.5 days in colostrum-fed calves [46], in contrast to IgA and IgM that have a half-life of only 2.8 and 4.8 days, respectively [47], which cause a more pronounced decline in time for the latter two. This could also explain the positive correlations between calf serum concentration traits with absolute amount of colostrum antibodies fed (Fig. 1). The IgG traits and STP had a slope of 0.45 while the IgA and IgM traits had a less pronounced one (0.32) because of their shorter half-lives.

Volume of the first colostrum milking and colostrum sampling time after calving are two important factors to consider when analyzing antibody concentration in the colostrum. We had data only for sampling time after calving which had a significant effect (negative) and was included in the model. Regarding volume of the first colostrum milking, some studies have shown a negative association with colostrum IgG concentration [11, 30, 44] and it is generally accepted that a larger volume creates a dilution effect. Not having these measurements is a limitation of our study, and future studies should include them.

Body condition score (BCS) at calving has been shown to be positively associated with health and milk production traits [48], but this factor is influenced by parity [49], age [50], calving season [51] and herd-related factors such as diet [49]. Notably, Denholm et al. [52] and Soufleri et al. [53] did not find a correlation between BCS and colostrum composition. Unfortunately, we did not have BCS data for our animals, but including parity, herd and calving season in our model, may account for some of the variation that could have potentially been explained by BCS. It is also worth noting that a permanent environment effect was included in the model, to account for constant environmental effects on the phenotypes across repeated measurements, which may have accounted for some additional environmental factors that influence BCS.

Other factors such as dry period length may have an effect, but the animals included in our study were subjected to a standard 8-week dry period so there is very little variation to measure an effect. In addition, Mayasari et al. [54] compared antibody levels in the colostrum from cows with a 0-, 30- and 60-day dry period and they found no difference between the cows with a 60- and 30- day dry period. Only the animals with a 0-day dry period had lower antibody levels in the colostrum. Andrée O'Hara et al. [55] found that IgG and total protein in the plasma did not differ between calves from cows with a 4- and 8-week dry period. Regarding age, we tested parity, age at first calving (AFC) and age at calving. Individually, all three were significant, but when including AFC + parity, only parity had an effect and the same was observed with age at calving + parity, so only parity was left in the model. Concerning dairy merit, it would not have been possible to include it in our model since 50% of the cows were first parity cows. The literature on this subject also shows that this factor does not always have an effect (e.g. MacFarlane et al. [56] found no association between the dam's previous lactation 305-day milk yield and passive transfer or colostrum IgG quality).

### Genetic correlations

#### Colostrum traits

Brix percentage was significantly and positively correlated with all the colostrum antibody traits, ranging from 0.49 to 0.73. Several studies have pointed out the use of Brix percentage to approximate the amount of antibodies in the colostrum [9, 57], namely IgG, but to the best of our knowledge this is the first report showing a genetic correlation with total IgG. Specifically, the correlation between Brix percentage and total IgG was 0.68, and even if the response to selection for Brix percentage is lower, it should be possible to collect more observations for this trait given its technical practicality and the possibility to measure it on farm compared to an ELISA or RID test (which measure total IgG directly), making it a promising indicator trait for selection of higher quality colostrum.

Apart from Brix percentage, total IgG was not genetically correlated with any other trait, but the correlation of 0.93 (0.23) with KLH-IgG was close to being significantly different from 0 (*p* = 0.06). The NAb traits showed a correlation pattern similar to that observed in a previous study of NAb in milk [32], where the IgM and IgA traits had very strong and positive genetic correlations between and within isotypes (0.82–0.97), suggesting a common genetic background for IgA and IgM as has been reported in milk by Wijga et al. [32]. The correlation between KLH-IgG and MDP-IgG was 0.76, which is high but sufficiently low to assert that they are different traits.

#### Calf serum traits

Unlike the colostrum, the indicator trait for calf serum (STP), did not have significant genetic correlations with any trait since the genetic variance and covariance of this trait were not significant. Although there is a slightly higher genetic correlation between traits from the same isotype and between IgA and IgM in calf serum, it is not as pronounced as in the colostrum. This implies that isotype may play a less important role for antibody uptake as previously observed by Burton et al. [13].

#### Colostrum traits versus calf serum traits

Measuring antibody absorption by the calf requires taking blood samples from the animal and then centrifuging them to separate the serum. This means that unlike the colostrum that can be sampled and measured on the farm (using a Brix refractometer), calf serum analyses require a veterinarian or technician for the sampling and a laboratory setting. For this reason, we wanted to estimate if colostrum quality in the cow correlates genetically with the calf’s ability to absorb antibodies. Originally, we attempted to correlate colostrum traits using cow as the genetic component with calf serum traits using calf as the genetic component, but there were convergence problems and correlations could not be estimated. Instead, we estimated these correlations using cow as the genetic effect on colostrum and calf serum traits. This is not ideal, but we wanted to get an idea of how these traits might correlate. Only the NAb IgA and IgM traits had significant genetic correlations within and between isotype traits. It seems that only the traits with very strong correlations could pass the significance threshold and even then, the values had large standard errors (0.13–0.29). Although most correlations were not significant, a positive trend was observed for some of them, such as colostrum Brix percentage and total IgG with calf serum IgG. A larger sample size is necessary to estimate these correlations more accurately, and to properly correlate the colostrum traits (using cow as the genetic effect) with the calf serum traits using calf as the genetic component, instead of cow as in this study.

### Production traits

#### Colostrum

To test if genetic selection for colostrum antibodies may have a negative effect on milk production, we estimated the genetic correlations with milk yield, fat and protein percentage, and lactation average somatic cell score for 305d lactation period following the calving at which the colostrum was sampled. We found no significant genetic correlations between colostrum traits and milk production traits, although the standard errors were rather high (0.13–0.28). Nonetheless, the results suggest that there are no strong genetic correlations. Total IgG tended to be positively correlated with fat and protein percentage. Brix percentage and protein yield seemed to have a positive trend, whereas Brix percentage and fat percentage had a slightly negative trend. The genetic correlation could not be estimated for Brix percentage and LASCS, but a phenotypic correlation of 0.12 (0.05) was found.

#### Calves

There is an unclear relationship between calf serum IgG (or FPT) and milk production performance later in life. Two of the studies that are usually referenced for this topic are: (a) Faber et al. [58] who found that calves fed 2 L of high quality colostrum at birth produced significantly less milk during the first and second lactations compared to animals that were fed 4 L, and (b) DeNise et al. [59] who reported that for every additional g/L of IgG in the serum of calves from 24 to 48 h of age, an increase of 8.5 kg of milk and 0.24 kg of fat was observed during the first lactation. However, these studies focus only on the phenotypes and do not look into the genetic components of these associations. To the best of our knowledge, the current study is the first to estimate genetic correlations between calf serum antibodies and milk production performance later in life. Our analyses did not find significant genetic correlations between calf serum traits and milk yield, protein percentage or fat percentage and the phenotypic correlations ranged from − 0.19 to 0.07 without a clear pattern.

Lactation average SCS is a log-derived measurement of SCC, a trait that is used as a surrogate for clinical mastitis and overall udder health. LASCS has a good genetic correlation with clinical mastitis (0.6–0.7) [60, 61]. In our study, we found significant negative genetic correlations between NAb and LASCS, i.e., KLH-IgM, MDP-IgM and MDP-IgA had correlations of − 0.98, − 0.96 and − 0.66 with LASCS, respectively. In addition, MDP-IgG, KLH-IgG and S-IgG had borderline significant negative correlations with LASCS. These findings suggest that NAb could be used to select for animals that are less prone to clinical mastitis, in agreement with the results of Thompson-Crispi et al. [18] for KLH-IgM and of Ploegaert et al. [62] for IgM and IgA NAb. Further analyses with a larger dataset are needed to confirm this association.

We did not find significant genetic correlations of calf serum traits with age at first calving (AFC). However, KLH-IgG had a borderline significant negative correlation with AFC (− 0.44), which is the desired direction, since increasing KLH-IgG would lead to a lower AFC. Age at first calving has been described as a proxy for average daily gain (ADG) since a higher value leads to an earlier insemination [2]. This result is in agreement with Furman-Fratczak et al. [63] who found that calves with a higher IgG level in their serum had higher growth rates, thus allowing for earlier inseminations.

## Conclusions

We have shown that all but one of the measured calf serum traits are heritable, including S-IgG, which indicate that genetic selection can be used to reduce FPT. Unfortunately, the indicator trait for calf serum, i.e., STP had a low heritability. Given the labor-intensive nature of the serological methods used to determine antibodies in the serum directly, an easy-to-measure trait such as STP was key to consider implementation in breeding programs. Interestingly, we found that there is a significant maternal contribution to calf serum antibody content in addition to the colostrum antibodies. Further analyses are needed to establish the genetic relationship between calf serum antibodies and milk production later in life. Regarding the colostrum, Brix percentage has positive genetic correlations with the investigated antibody traits, thus Brix percentage can be used as an indicator trait to select for a higher quality colostrum. In addition, our results suggest that Brix percentage may not be genetically correlated with milk production traits in an unfavorable way, but further studies with a larger dataset are needed to confirm this. This study showed that these traits can be used for selection programs that focus on improving genetically antibody content in both the colostrum and calf serum, pending a practical indicator trait for calf S-IgG.

## Data Availability

Some of the data that support the findings of this study were provided by Växa Sverige but restrictions apply to the availability of these data, which were used under license for the current study, and so are not publicly available. Data are however available from the authors upon reasonable request and with permission of Växa Sverige.
